# A Novel Experimental Apparatus for Characterizing Flow Regime in Mechanically Stirred Tanks through Force Sensors

**DOI:** 10.3390/s24072319

**Published:** 2024-04-05

**Authors:** Miguel Magos-Rivera, Carlos Avilés-Cruz, Jorge Ramírez-Muñoz

**Affiliations:** 1 Electronics Department, Autonomous Metropolitan University, Av. San Pablo 420, Col. Nueva el Rosario, Mexico City C.P. 02128, Mexico; mrm@azc.uam.mx (M.M.-R.); caviles@azc.uam.mx (C.A.-C.); 2 Energy Department, Autonomous Metropolitan University, Av. San Pablo 420, Col. Nueva el Rosario, Mexico City C.P. 02128, Mexico

**Keywords:** force sensors, pressure fluctuations, Reynolds number, flow regime, mixing tank

## Abstract

Pressure fluctuations in a mixing tank can provide valuable information about the existing flow regime within the tank, which in turn influences the degree of mixing that can be achieved. In the present work, we propose a prototype for identifying the flow regime in mechanically stirred tanks equipped with four vertical baffles through the characterization of pressure fluctuations. Our innovative proposal is based on force sensors strategically placed in the baffles of the mixing tank. The signals coming from the sensors are transmitted to an electronic module based on an Arduino UNO development board. In the electronic module, the pressure signals are conditioned, amplified and sent via Bluetooth to a computer. In the computer, the signals can be plotted or stored in an Excel file. In addition, the proposed system includes a moving average filtering and a hierarchical bottom-up clustering analysis that can determine the real-time flow regime (i.e., the Reynolds number, Re) in which the tank was operated during the mixing process. Finally, to demonstrate the versatility of the proposed prototype, experiments were conducted to identify the Reynolds number for different flow regimes (static, laminar, transition and turbulent), i.e., 0≤Re≤ 42,955. Obtained results were in agreement with the prevailing consensus on the onset and developed from different flow regimes in mechanically stirred tanks.

## 1. Introduction

Mixing operations are of significant relevance in many manufacturing and production processes commonly used in the industry, impacting the final product’s characteristics and overall success. They can involve a single phase, with the goal of achieving temperature homogenization in the product, or multiple phases, aiming to disperse and uniform the concentration of various ingredients. Thus, this operation can be found in the production of food and beverages [1], in the production of polymers [2], in the preparation of paints [3], in chemical and petrochemical processes [4] and in the preparation of pharmaceuticals [5], among others. Process parameters, such as process time, energy consumption or the quality of the final product can be directly related to a correct mixing process [6]. Commonly, this process is carried out in cylindrical tanks with stirrers driven by electric motors. The type of paddle, as well as its position, angle of inclination, size and thickness, are some of the characteristics that have been studied in order to have agitators that are better adapted to the different mixing processes required by the industry [7]. The influence of the shape of the vessel on this process has also been studied [8]. It is a common practice to introduce baffles in the tank walls to improve mixing efficiency above the transitional flow regime [9].

Depending on the final objectives and whether the mixing process is limited by shear (e.g., dispersion of pigment particles, bubbles and droplets) or pumping (e.g., blending of miscible liquids, heat transfer or solid suspensions), as well as the working fluid viscosity, the mixing process in stirred tanks will be optimally performed in the laminar, transitional or turbulent regime. For instance, for pigment dispersion, as TiO_2_, into water for the elaboration of slurries, the utilization of high-shear impellers operating at Re in the laminar regime at relatively high speeds, exhibits a superior performance compared to transitional and turbulent regimes [10,11]. On the other hand, mixing efficiency in processes limited by pumping with conventional impellers (e.g., Rushton, pitched blade or high efficiency) is more effective in the turbulent regime [12,13].

At the industrial level, the measurement of the parameters involved in the mixing operation can be useful in a closed-loop control scheme to ensure the correct operation of the process or for monitoring tasks, which are becoming very important with the emergence of the Industry 4.0 concept. Likewise, much of the research work related to this operation requires the measurement and acquisition of some of the variables involved. This allows researchers to carry out analyses and simulations of the behaviour of the elements in particular and of the process as a whole, with the aim of designing better equipment that increases the efficiency of production processes.

This paper presents the design and construction of a Monitoring and Acquisition System for Pressure Fluctuations in Stirred Tanks (MASPFST). As its name suggests, the equipment built enables the recording, at the laboratory level, of variations in pressure (force/area) that occur at specific points on the walls or baffles of a stirred tank. Using Force Resistive Sensors (FSR), an electronic system based on a microcontroller collects data from up to five sensors. The information is stored locally during the experiment and then transmitted wirelessly to a computer for final storage. In this way, the device is presented as a tool that will allow researchers to monitor pressure fluctuations in the mixing tanks.

Due to its importance at the industrial level, a large number of works have been oriented towards the development of equipment for the measurement and data acquisition of variables involved in mixing processes. In [14], the authors describe the use of ultrasonic sensors to measure particle concentration in a solid-liquid mixing process. The measurement is made by determining the change in power of an ultrasonic signal as it passes through the mixture being analyzed. The sensor signal is read on an oscilloscope connected to a computer where the information is stored. Similar investigations can be found in [15,16]. Another parameter whose measurement in mixing systems has been extensively studied is the concentration, which is directly related to the mixing time. The use of colorimeter for determining the uniformity of a mixture by using digital colour imaging method (DCI) and comparing it with a salt conductivity method is described in [17]. The authors conclude that it is an economical method with a good degree of accuracy. On the other hand, Ref. [18] presents a methodology based on conductivity to perform a homogeneity study in mixtures. They reported that this technique works adequately with conductive liquids as water. Several studies have been carried out using electrical resistance tomography to determine the performance of mixers [19,20].

Ultrasonic sensors, while effective for non-contact measurements and applicable in fluid environments, encounter challenges in accurately gauging turbulent conditions, especially in stirred tanks where rapid turbine blade movement disrupts measurements. The unpredictable scattering and reflection of ultrasonic waves in such environments undermine measurement reliability and risk signal interference. Conductive sensors and electrical resistance tomography techniques are not suitable for use with fluids commonly employed in the paint and coating industry, such as alkyd or epoxy resins, which typically exhibit low electrical conductivity. DCI requires a significant investment in specialized equipment and software. Additionally, the installation, calibration and maintenance of these systems can be complex and require trained personnel.

Recently, a novel method [21] was proposed for identifying the flow regime (flooding, loading and complete dispersion) in an aerated stirred tank with a Rushton turbine by using pressure fluctuation signals. Statistical, Hurst, Shannon entropy and chaos analysis methods were used to analyze the pressure time series. In other studies, the flow characterization in aerated tanks are based on changes in the gassed power draw [22] or combining the power draw with visual observations [23]. Measurements of the flow velocity in pipes with single- and multi-phase using electrical capacitance tomography and pressure sensors have been reported [24,25].

While the aforementioned technologies show promise in enhancing mixing processes and characterizing flow in aerated systems, their successful integration into stirred tanks and their effectiveness in determining flow regimes with homogeneous fluids require further research to validate their applicability across a wide range of Reynolds numbers, spanning from laminar to fully turbulent flow regimes. The analysis of the state of the art does not report any work concerning the real-time identification of the flow regimes (static, laminar, transition and turbulent) in mechanically stirred tanks using Force Resistive Sensors. They provide direct measurement of mechanical forces exerted by the fluid flow onto the tank walls or sensor surfaces and can be used with both conductive and non-conductive fluids, as well as with corrosive or abrasive substances commonly found in industrial processes. Traditional method to identify the flow regime involve obtaining the offline impeller power curve (power number versus Reynolds numbers) that requires a complete set of power measurements (electrical or based on torque) [26], and additionally, measurements of the density and viscosity of the mixing fluid. However, obtaining real-time data for these parameters poses challenges in practical applications.

This paper presents the design, construction and performance tests of a prototype device that allows the characterization of the real-time flow regime in mechanically stirred tanks by monitoring the pressure fluctuations. Notably, this method eliminates the need for prior knowledge of fluid density and viscosity. A series of experiments in a tank stirred with a standard Rushton turbine and equipped with four vertical baffles were performed to verify the correct operation of the system.

The paper is organized as follows. In Section 2, general equipment description is presented. In Section 3, results are described, and finally, in Section 4, conclusions and future works are given.

## 2. Design of the Apparatus

**Figure 1:**
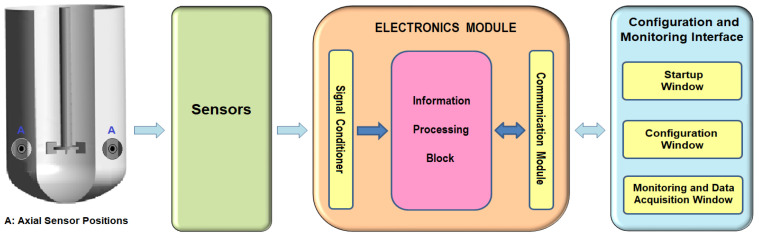
General scheme of the pressure fluctuation detection system in stirring tanks.

The MASPFST senses the pressure (force/area) behavior at specific points in a stirred tank and stores the information in data files for further analysis. In addition to the sensors, the system consists of an electronic module and a configuration and monitoring interface. To avoid exposing the computer equipment to splashes during experiments and to reduce the number of cable connections in the work area, information is transmitted wirelessly between the electronics module and the computer using the Bluetooth communication protocol. This allows the computer equipment to be located up to 10 m away from the experiment site. Figure 1 shows a schematic of the complete system and the axial sensor positions within the tank.

The key features of the three blocks that make up the system are presented in the following subsections of this chapter.

### 2.1. Sensor Features

**Figure 2:**
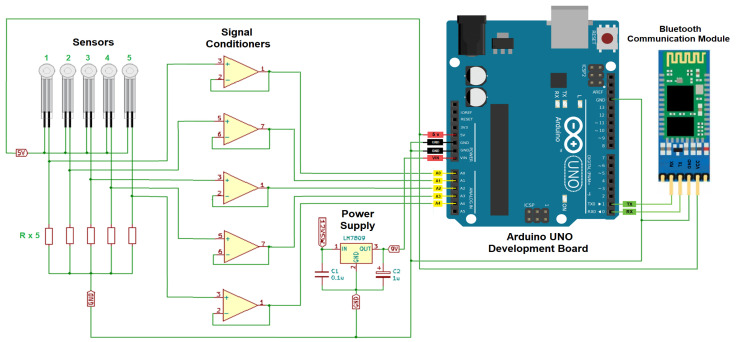
Connecting the signal conditioner, sensors, Bluetooth communication module and power supply to the Arduino UNO board.

A Force Sensing Resistor (FSR, serie specification FSR04), from Ohmite© Manufacturing Company (Brownsville, TX, USA) was selected to monitor pressure variations at the points of interest in the stirred tank. This is a tactile type device commonly used to detect pressure in robot grippers, keyboards, buttons, etc. The advantage of these elements is that they are small compared to traditional load cells. They are usually made of flexible material, which allows them to be thin and light. In this prototype, Ohmite’s FSR 04 model was chosen. It is a piezo resistive sensor placed between two sheets of flexible polyester whose thickness is less than 0.5 mm. The sensors are shown in the electrical diagram of the electronic module in Figure 2 (left-side).

This sensor has a linear relationship between its electrical conductance and the applied pressure (force/active area). Without pressure on the element, the resistance is very high: about 10 MΩ. When the equivalent of 5 kg is applied on its active area of 5.6 ${\mathrm{mm}}^{2}$, the sensor presents a resistance in the order of 5 kΩ. Tests were conducted by applying various hydrostatic loads to one of the sensors. The results obtained align with the specifications provided by the device manufacturer. It is important to mention that this type of element is generally used to detect pressure variations, therefore the calibration values are not accurate. The above is adapted to the equipment built, since the exact value of the pressures in the tank is not of interest, but rather the way in which they vary under the different flow regimes throughout the mixing process.

### 2.2. Electronics Module

The electronics module contains electronic devices that are responsible for conditioning, processing and transmitting the information generated by the sensors placed in the stirring tank. To facilitate the explanation of the structure of the electronics module, a block diagram is shown in Figure 1 (third figure). As can be seen, it consists of three main parts: a Signal Conditioner, Information Processing Block and Communication Module, which are described in detail in the following sections.

#### 2.2.1. Signal Conditioning

The first block of the electronics module is called Signal Conditioner. Its function is to prepare the signals sent by the sensors to be used by the rest of the system, in this case the information processing block. For the construction of the prototype described in this document, the sensors selected are of the resistive type, that is, the resistance of the element varies when there is a change in the pressure exerted on it. Taking this into account, the conditioning circuit designed and built for the sensors is a voltage divider complemented by a voltage follower implemented with an operational amplifier. In this way, the resistance variations of the sensors are converted into voltage variations that are sent to the next block of the electronics module. Figure 2 shows the electrical diagram of the conditioning circuit for the five sensors.

From the information provided by the manufacturer and from tests carried out on the sensors, it was determined that the resistance of the element varies between 10 MΩ with no pressure applied and 10 kΩ with a load equivalent to 3 kg. Taking into account the nature of the application, it was considered that it would be difficult to have higher pressures than those indicated, so the values indicated were taken as a starting point for the calculation of the electronic circuit. In addition, the sensor manufacturer recommends the use of no more than 6 V DC with the current not exceeding 1 mA. Based on the above restrictions, a maximum output voltage of 4 V DC and a maximum current of 0.1 mA are suggested for a 3 kg of load. The equation for the voltage divider is given in Equation (1).

(1) $$V_{O\_max}=\frac{R}{R+R_{sensor\_min}}V_{i},$$

where

$V_{O\_max}$: Maximum output voltage, default 4 V DC.

$V_{i}$: Power supply voltage, 5 V DC.

$R_{sensor\_min}$: Minimal resistance of the sensor, 10 kΩ.

*R*: Resistance to be determined.

Subtract and replace to obtain the resistance value: *R* = 40 kΩ. The commercial value of 39 kΩ is suggested. It is verified that with this value the maximum current in the voltage divider circuit does not exceed 0.1 mA. Assuming that the output current of the circuit is practically equal to 0 mA, the maximum current in the $I_{max}$ resistive circuit is given in Equation (2).

(2) $$I_{max}=\frac{V_{i}}{R+R_{sensor\_min}}=0.1\;\mathrm{mA}.$$

It is observed that in the extreme case, with a minimum resistance of 10 kΩ in the sensor, the established value of 0.05 mA for the current in the circuit is not exceeded. The power of the resistor was chosen to be 250 mW, a value that has a fairly wide margin considering the voltage and current in the voltage divider circuit. The operational amplifier used for the signal conditioner was the LM358 (from National Semiconductor, Santa Clara, CA, USA), this 8-pin integrated circuit contains two general purpose amplifiers. The prototype built has five conditioning circuits, one for each sensor.

#### 2.2.2. Communication Module

This block of the electronics module is responsible for transmitting the information acquired by the equipment to the computer that monitors and stores the data obtained by the sensors. Due to the conditions in which the experiments are carried out in practical applications, it was considered to avoid the use of cables to transmit the information between the electronics module and the computer. Therefore, the Bluetooth wireless communication standard was chosen. This method of information transfer uses radio waves in the 2.4 GHz frequency, which corresponds to the ISM band. Although the range is limited (maximum 10 m), it avoids the use of cables and does not require line of sight between the devices to communicate, meeting the system’s specified requirements.

The HC-06 Bluetooth communication module is depicted in the electrical diagram of the electronic module shown in Figure 2. It is an integrated electronic system that complies with the specifications of the Bluetooth 2.0 standard, which allows it to communicate with smartphones, computers, tablets, as well as digital systems that handle this protocol. The data to be transmitted is provided to the board in serial UART TTL form, the module is pre-configured at the factory to function as a slave operating at a rate of 9600 bps. Its dimensions are 37 × 16 mm and weight 3.2 g. The HC-06 communication card has 4 terminals located on one side of the card.

The function of each signal handled by the HC-06 module is described below.

RX: This is the receiving terminal, where the information processing block sends the data to be transmitted via Bluetooth to the computer through the serial port.

TX: Through this terminal, the HC-06 module sends to the information processing block the data received by Bluetooth from the computer.

GND: This terminal is used to connect the power supply reference.

VCC: This terminal is used to supply power to the module for its correct operation. The power supply range can vary between 3.3 and 6 V DC, with a maximum current of 40 mA.

#### 2.2.3. Information Processing Block

The information processing block is the central part of the electronics module. It is the system that receives the conditioned signals from the sensors installed in the tank. This block receives the information and organizes it to be delivered to the communication module, which is responsible for sending it to the computer equipment. The central element of this block is a commercial board type Arduino model UNO; this device has, among other features, six analog input channels. Since there are five sensors that are handled in the prototype described here, the selected model of the Arduino board covers the needs of the prototype. The description of the information processing block is divided into two sections. The first section is related to the physical connections of the elements (signal conditioners and communication module) with the Arduino board. The second part corresponds to the description of the program developed for the digital system; both parts are described in detail below.

Electrical connections for Arduino UNO Board. As mentioned above, the information processing block is based on an Arduino UNO development board. It is a digital system built around an ATmega 328 microcontroller (from Atmel Corp., San José, CA, USA).

The board has 1 kB of EEPROM memory, digital input and output ports and analog input ports. It also has serial communication ports for exchanging information with other devices, power inputs and outputs and a USB port for connecting to the computer to transfer the microcontroller program.

Table 1 shows how the signals used in the Arduino UNO were connected in the application described in this paper.

Analog inputs. Of the six analog inputs on the Arduino UNO board, five of them were used for the connection of the same number of sensors. However, only four sensors (one in each baffle) were used in the mixing tank experiments. The 5 V DC signal for the voltage dividers of the signal conditioners was also taken from the development board. Figure 2 (left-side) shows the electrical circuit with the described connections.

Serial port. In the case of the serial communication terminals of the Arduino UNO board, they were used to exchange information with the Bluetooth communication module HC-06. The connections between these devices are cross-connected: the TX terminal of the Arduino UNO is connected to the RX of the HC-06, while the RX signal of the Arduino UNO is connected to the TX terminal of the Bluetooth module. Figure 2 (right-side) shows the connection diagram between the elements.

Power input. The last set of terminals used from the Arduino UNO development board is the one associated with the power supply of the device. A voltage of 9 V DC was used to power the electronic board. Since the system as a whole is powered by a 12 V DC supply, an LM7809 voltage regulator was used to generate the voltage required for operating the Arduino UNO board. Figure 2 shows the connection diagram between the elements.

**Figure 3:**
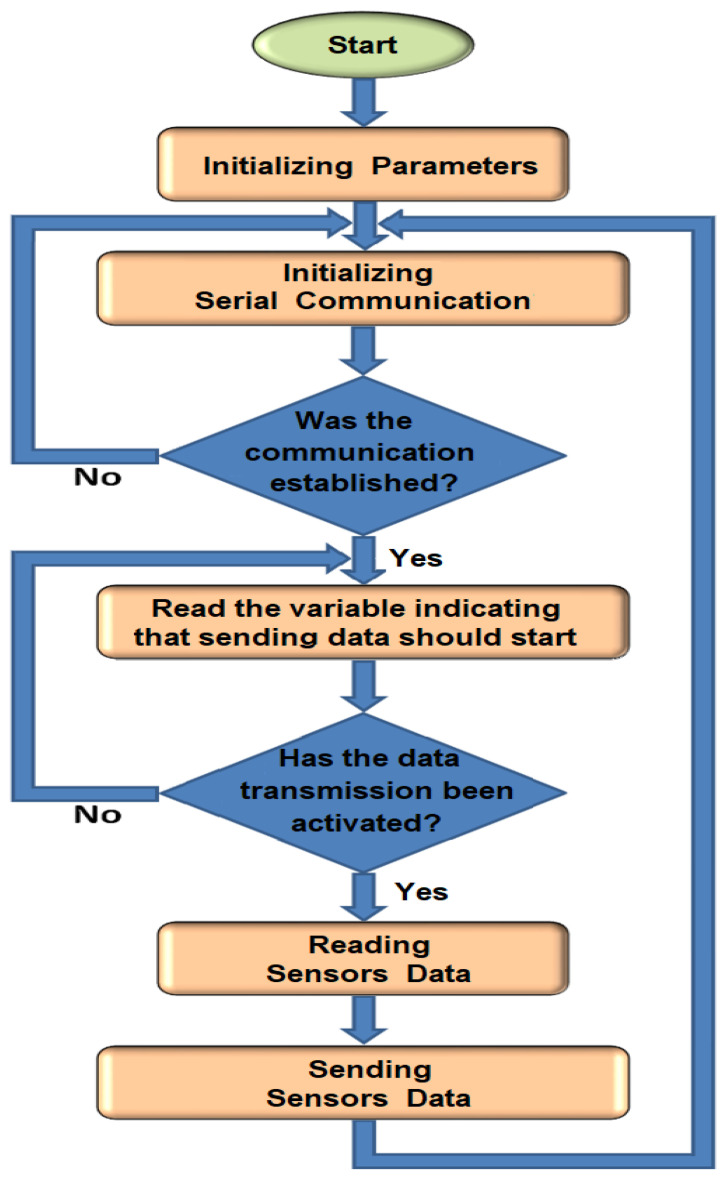
Program flowchart for Arduino UNO board.

Arduino UNO programming. This section of the document describes in detail the program developed for the Arduino UNO board. Figure 3 shows the general flowchart of the tasks performed by the program, which are described below, along with the code developed.

Initializing Parameters. In this first part of the program, the Arduino UNO terminals are assigned to the analog inputs that receive the signals from the sensors. In addition, two variables are declared that will be used to signal the start of reading and sending data. The serial communication between the Arduino UNO and the HC-06 module that will transmit the information to the computer is initialized. It is determined that the communication will take place at a rate of 9600 bps. The code section is shown in Table 2.

Readout of sensor values and transmission via Bluetooth. The Arduino UNO checks if communication with the computer has been established. If so, it proceeds to the next section, otherwise it waits. After establishing communication between the electronics module and the computer, the Configuration and Monitoring Interface sends a variable indicating that the data transfer must be initiated. This is accomplished by pressing the Start button in the Data Capture box of the Monitoring and Data Acquisition window within the interface. The next step is to check the value of the read variable that indicates whether the data transfer should start. If the transfer should start, a flag is set to indicate this and the rest of the program continues. Otherwise, the device waits for a positive response.

Read analog ports. The digital system reads the conditioned signals from the sensors connected to the analog ports of the Arduino UNO.

Send data. Finally, the conditioned signals from the sensors are sent through the serial port to the Bluetooth communication module for transmission to the computer.

### 2.3. Configuration and Monitoring Interface

The system is equipped with a program developed in Visual Basic 15.5, which serves as a user interface. There are three windows that make up this application: Startup Window, Configuration Window and Monitoring and Data Acquisition Window. The following sections describe the characteristics of each of these windows.

#### 2.3.1. Startup Window

**Figure 4:**
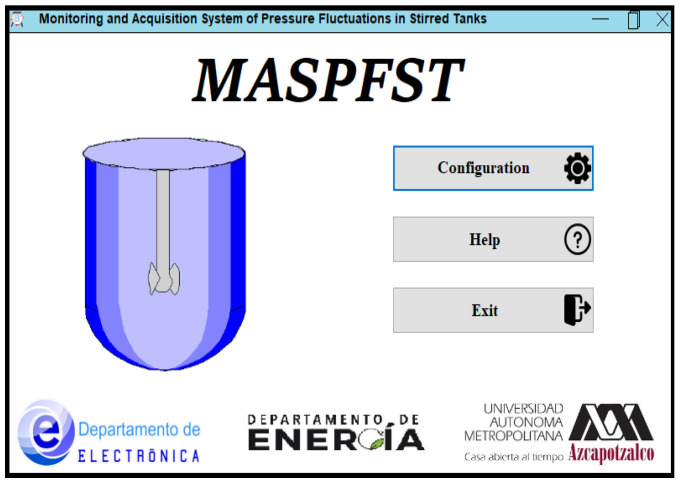
Configuration and Monitoring Interface: Startup Window.

When the application is launched, the Start Window appears, with three buttons in the middle. The first two correspond to the Help and Exit options, the third button allows access to the window in which the system parameters necessary for monitoring and data acquisition of the experiment are configured. The title and acronym of the developed system are displayed at the top of the main window: Monitoring and Acquisition System of Pressure Fluctuations in Stirred Tanks, MASPFST. In the lower part of the screen there are the logos of the institution and the departments involved in the development of the system, the construction of which is described in this document. The Startup Window of the application is shown in Figure 4.

#### 2.3.2. Configuration Window

When the Configuration button is clicked, a new window appears in which is possible to set the parameters required by the instrument to perform the monitoring and acquisition of the experimental data. In addition to the configuration of the experiment, this option includes a button that gives access to the functions related to the monitoring, acquisition and storage of the acquired data. Pressing the Help button displays a PDF file of the system user manual.

**Figure 5:**
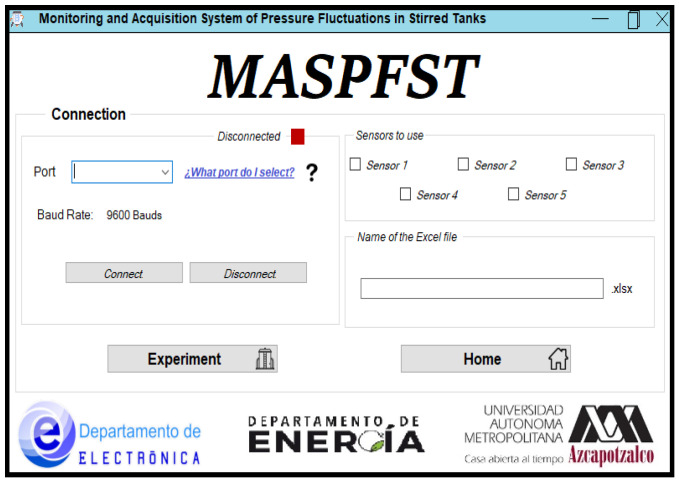
Configuration and Monitoring Interface: Configuration Window.

Parameters that determine how the system operates can be set in the Configuration Window. There are three sections that make up this screen: Connection, Sensors to be used and Excel file name. In addition, there are two buttons: the first allows access to the functions related to monitoring and data acquisition, while the second takes the user to the application start screen. It should be noted that as long as there is no communication with the electronics module (Connect button), the Disconnect and Monitoring and Data Acquisition buttons are disabled. Figure 5 shows a view of the parameter Configuration Window of the Configuration and Monitoring Interface.

Connection. This block groups the parameters related to Bluetooth communication between the computer and the electronics module. There is a field where the user can specify which port of the computer will be used to communicate with the electronics module. In addition, there is a label with the speed at which the devices will communicate, an indicator, this time luminous, will indicate whether the interface is connected to the module or not. Finally, there are two buttons to initiate or terminate the connection between the devices. Figure 5 shows the elements in the Connection block of the MASPFST interface configuration window. On the upper-left there is a port configuration zone, i.e., the COM 05 communication port, operating at 9600 baud and without connection, while on the right there are the same parameters, but this time the system is already connected to the electronics module.

Sensors to use. The elements included in this block of the configuration window are five selection boxes, one for each sensor. Here the user can tell the system which sensors are to be monitored and stored. Figure 5 (upper-right zone) shows the distribution of items in the Sensors to use block of the Configuration Window of the MASPFST interface.

Name of the Excel file. In this block, the user specifies in the text box the name of the Excel file in which the data collected during the experiment will be stored. Figure 5 shows this block (middle-right zone).

#### 2.3.3. Monitoring and Data Acquisition Window

**Figure 6:**
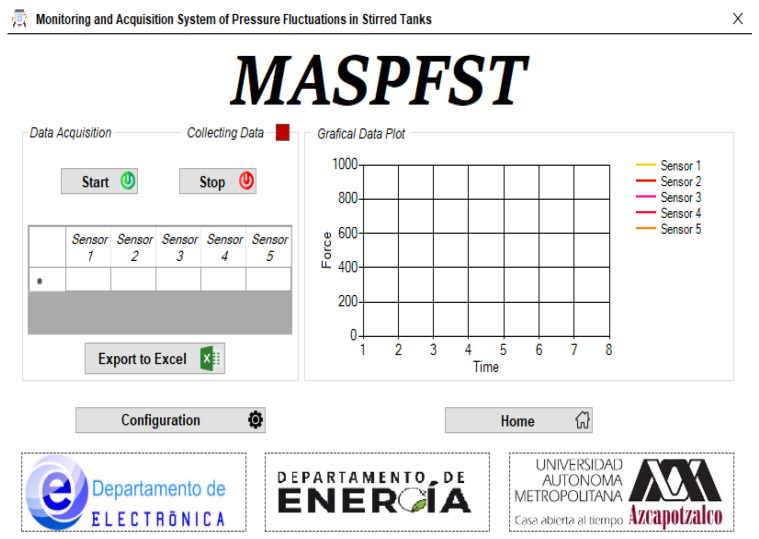
Monitoring and Data Acquisition Screen of the Interface.

The last of the windows that make up the MASPFST Configuration and Monitoring Interface is the one related to the process monitoring and data acquisition options. The screen is divided into two sections: Data Acquisition and Data Graph. There are also two buttons, the first allows the user to return to the Configuration Window, while the second takes the user back to the start of the application. Figure 6 shows a view of the Monitoring and Data Acquisition Window of the Configuration and Monitoring Interface.

Data Acquisition. This block contains two selectors, which are used to start and stop the monitoring and data acquisition of the current experiment. The activation of these selectors is interdependent, i.e., if one is activated, the other is deactivated. At the start of monitoring and data acquisition, the last data acquired by each sensor are displayed in numerical form in the table contained in this block. An indicator is also displayed at the top to indicate that the system is collecting data. The last element of this block is the button that the user must press to start sending the data read by the device to the Excel file whose name has been defined in the Configuration Window. The Export to Excel button is disabled when no data is stored or when data acquisition is in progress.

Once the data collection is stopped, the Export to Excel button is enabled. When clicked, it initiates the transfer of information to the Excel file whose name was specified in the Configuration Window.

Data Graphing. This section of the Monitoring and Data Acquisition Window graphically displays the last 8 s of variation from the force sensors placed in the stirred tank. The graphs are displayed only for the sensors specified in the Configuration Window. Similarly, the graphs of the variations of the variables of interest are displayed only when data acquisition is enabled. Figure 6 (right-side window) shows the graph block of the Monitoring and Data Acquisition Window.

## 3. Apparatus Construction

**Figure 7:**
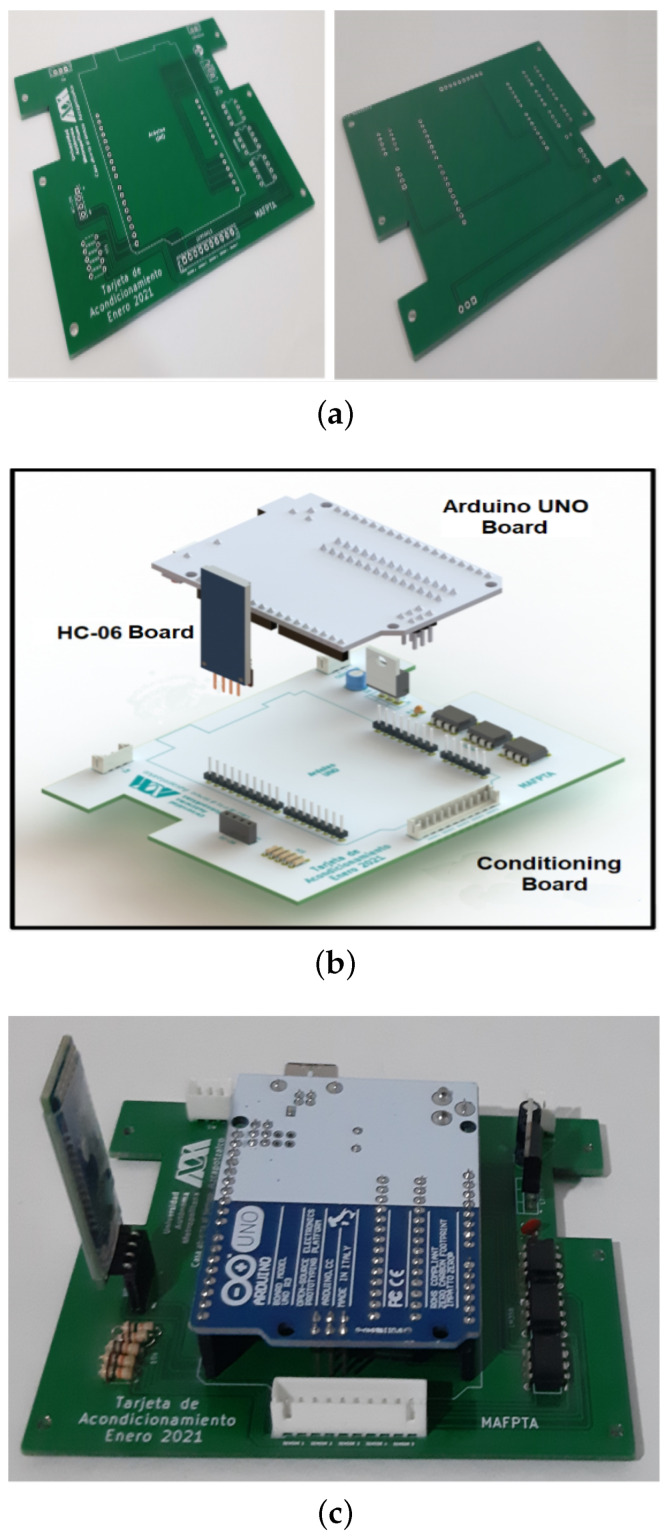
Electronic printed circuit boards. (**a**) Electronic built card. (**b**) Perspective view of the three components of the electronic cards. (**c**) View of the assembled electronic printed circuit boards.

A printed circuit board designed and built for this application contains the elements that make up the electronics module. The electronic devices associated with the signal conditioners, as well as those that regulate the electrical power that feeds the equipment, are connected to this board. The Arduino UNO development board and the HC-06 communication module are also installed on the board, which, in addition to serving as a support, allows the interconnection of the blocks that make up the electronics module. Figure 7 shows images of the three electronic boards that make up the Electronics Module. First, there are views of the two sides of the built board card, then an exploded view of the three boards and, finally, their assembly.

The electronic cards described above are housed in a Serpac Model 173-Almond plastic enclosure (from Serpac Electronic Enclosures, La Verna, CA, USA).

**Figure 8:**
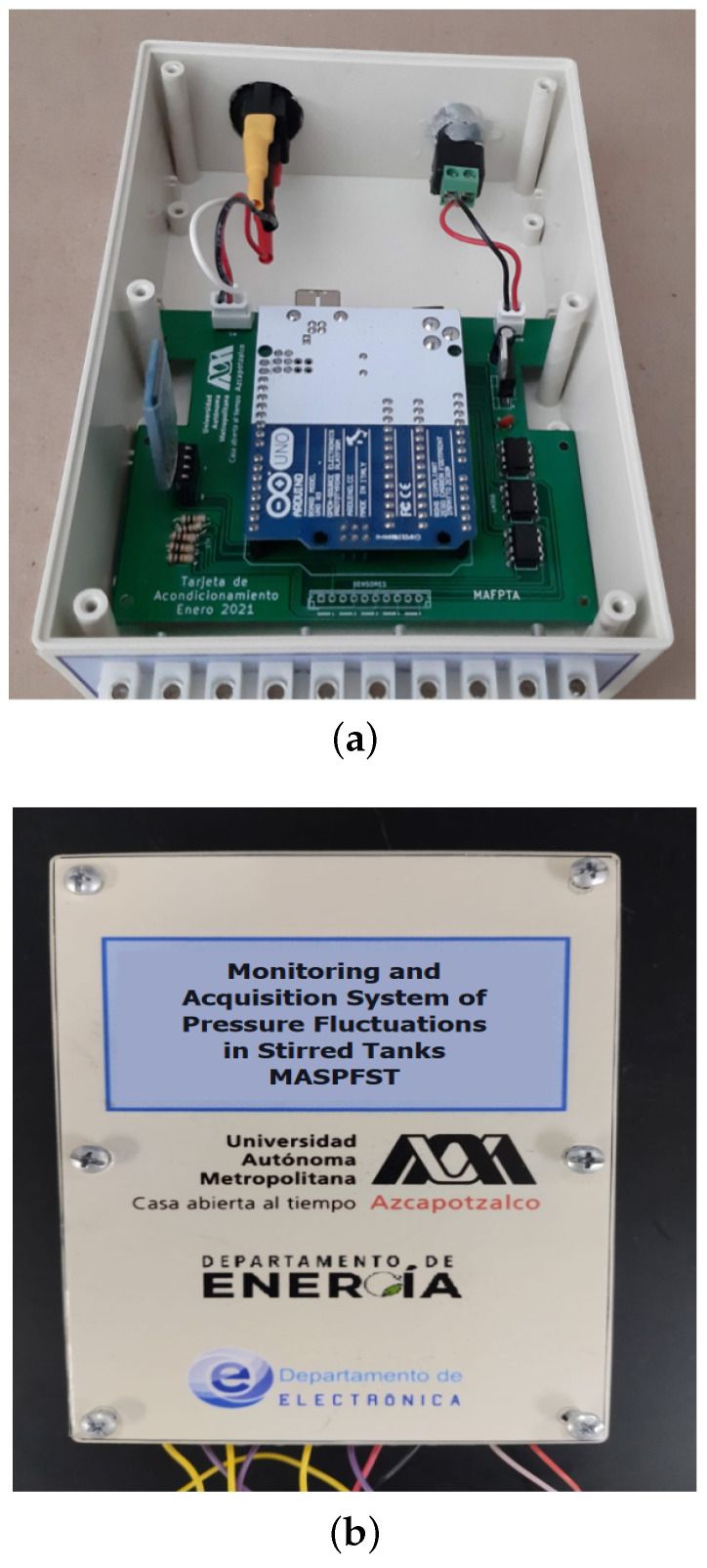
Electronics module of the system. (**a**) The inside of the cabinet with the boards installed. (**b**) The final prototype.

It is a box with a lid with the dimensions: 175×124×64 mm and a weight of 250 g. On the front of the case there is a 10−pin connector for the five force sensors, while on the back there is the instrument’s power switch and the power supply connector. The unit requires a 12 V DC power supply with a minimum of 2 A. Figure 8a shows the inside of the cabinet with the electronics boards installed, while Figure 8b shows the final prototype.

## 4. Performance Tests

The proposed system was realized with a mixing tank provided by four vertical baffles, and a force sensor was installed on each baffle. Sensors were located in baffles at the same height of the flow induced by the used radial flow impeller (Rushton type). To avoid heat losses in some experiments that require heating, the external wall tank was covered by a thermal insulator.

### 4.1. Baffle and Sensors

**Figure 9:**
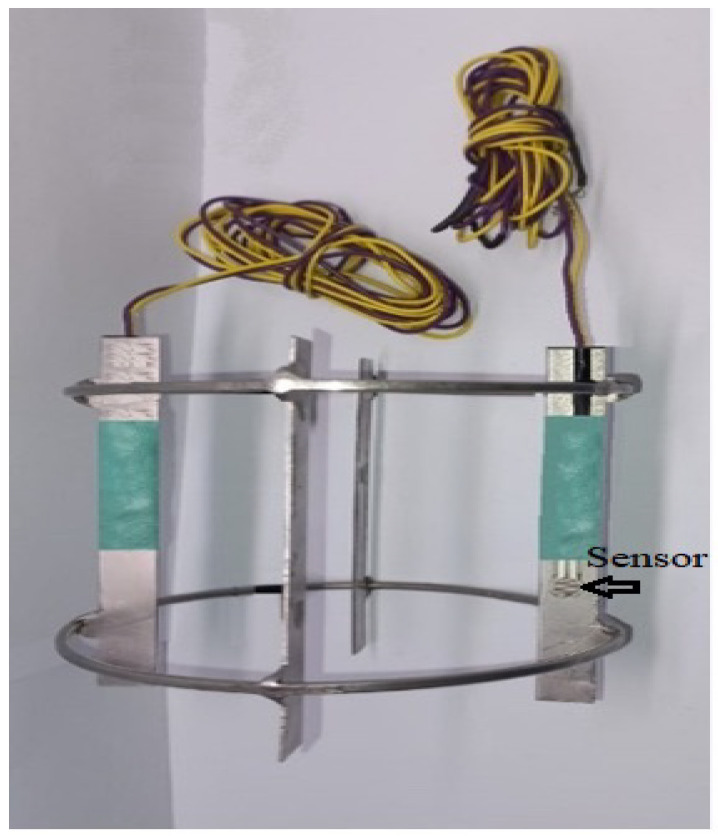
Baffles and force sensors.

In order to improve the mixing efficiency inside the tank, a removable baffle system with four sensors equally spaced is included. The height of each baffle is similar to the tank diameter, its width is equivalent to 1/10 of the tank diameter, while the thickness of the stainless steel plate used to made the baffles was 3.2 mm. This element adapts to the inner diameter of the tank, and the force sensors have been installed on the baffle as shown in Figure 9.

### 4.2. Monitoring and Acquisition System

The four sensors placed on the baffles were connected to the same number of inputs of the electronics module of the Pressure Fluctuation Monitoring and Acquisition System. On the other hand, the parameters of the Configuration Window of the Configuration and Monitoring Interface of the system were configured according to the data contained in Table 3.

### 4.3. Agitation System and Mixing Tank Experiments

Agitation was generated by a 0.75 HP (560 W) variable-speed Dispermat®AE01 motor manufactured by BYK-Gardner GmbH, Geretsried, Germany. This equipment includes, in addition to the motor, a speed controller with an operating range of 0 to 10,000 rpm. The impeller is a standard Rushton turbine with six blades and a diameter of 50.8 mm. Detail dimensions of the tank and impeller used in this study can be found in a paper published by Ramirez et al. [27].

**Figure 10:**
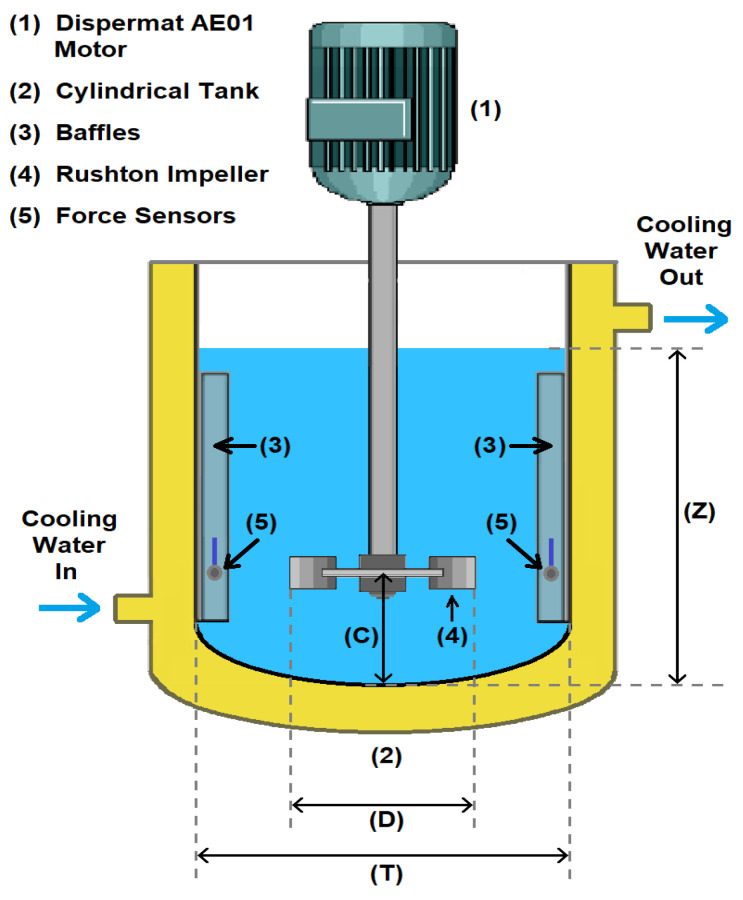
Mixing tank experimental setup.

Experiments were conducted within a baffled, cylindrical stainless steel jacketed vessel featuring with a dished bottom and internal diameter T = 132 mm (see Figure 10). The dimensionless geometric ratios associated with the impeller’s off-bottom clearance (C), the liquid height (Z) and the impeller diameter (D) were as follows: C/T=0.3848, Z/T=1 and D/T=0.3848, respectively. Four sensors were used in the experiments for recording the pressure fluctuations (namely, Sensor 1, Sensor 2, Sensor 3 and Sensor 4, respectively). The sensors were located in the middle axial distance of the baffles with an angle between them of $90^{\circ }$ and were oriented towards the impeller discharge radial flow. They were connected by wires with the monitoring and acquisition system and the data are wirelessly transmitted to the PC. Wireless data transmission proves to be invaluable in supervisory control and data acquisition (SCADA) systems, facilitating the collection and analysis of real-time data pertaining to crucial and time-sensitive information. For instance, in mixing tanks, it plays a crucial role in preventing runaway reactions.

For covering the impeller Reynolds number ($Re=\rho \cdot N\cdot D^{2}/\mu$) range from laminar to fully turbulent flow investigated in this study, two Newtonian solutions of different viscosities (μ) and densities (ρ) were used, and in addition, the Rushton impeller speed (*N*) was varied in the range between 25 and 1100 rpm. These consisted of two solutions prepared from a $45^{\circ }$ Bx food grade glucose and distilled water at mass glucose concentrations of 20% (fluid 1) and 0% (distilled water, fluid 2), respectively. Table 4 shows the properties of the two employed fluids and the impeller Reynolds number covered for each fluid at 20 °C. The viscosities were determined using an Anton Paar MCR 502 rheometer with a concentric cylinder geometry, while their corresponding densities were measured using a graduated cylinder and an analytical balance. A cooling bath was employed to maintain the fluid process temperature at 20 °C ± 0.5 °C.

### 4.4. Analysis of Pressure Fluctuations

The prevailing consensus in the literature is that for Reynolds numbers (*R*e) ≥ 10,000, the flow inside a baffled stirred tank can be considered as predominantly turbulent and the creeping laminar flow regime is limited to Re around 10 [13,28], whereas at 10 < Re < 130, the interaction between the impeller-induced flow and the baffles is still weak [29]; therefore, this Re range can be considered as extended laminar flow. On the other hand, the flow between the above Reynolds numbers can be considered in the transitional flow regime [30].

As an application example of the MASPFST, this system was used to detect the flow regime inside the tank by using two pressure fluctuation measurements (Sensors 1 and 3) located in the baffles. The two tested fluids were run at different Reynolds numbers by varying the impeller speed across eleven flow conditions, as follows: Re=0 (static), Re=85, Re=128, Re=1160, Re=2148, Re=8591, Re= 17,182, Re= 25,773, Re= 34,364 and Re= 42,955. For each Reynolds number condition, the four sensors were recorded simultaneously for an acquisition time of 70 s at a sampling frequency of 10 Hz, and the data were stored in an Excel file.

**Figure 11:**
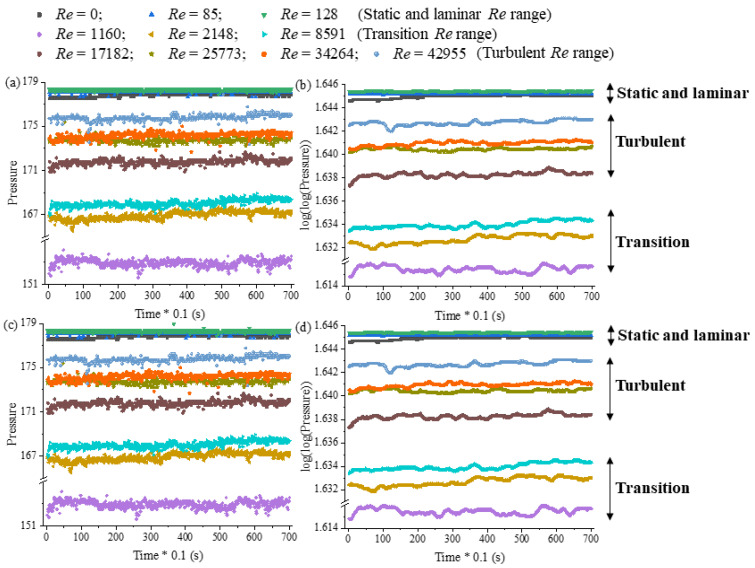
Displaying the data acquired by the system. Sensor 1: (**a**) raw data and (**b**) filtered data. Sensor 3: (**c**) raw data and (**d**) filtered data.

The recorded data were analyzed using data preprocessing techniques and obtained results for Sensors 1 and 3 are showed in Figure 11. First, the data were plotted as recorded “raw data” by plotting time versus pressure for the eleven Reynolds numbers studied (see Figure 11a,c). Next, double log base 2 and moving average filtering (average of 20) were applied and plotted (as shown in Figure 11b,d). The employed moving average filtering relies on statistical averages, necessitating the definition of the number of samples to be averaged. Through experimentation, it was determined that a minimum of 20 samples should be averaged. A smaller sample size exhibited variations in the signal resembling the original, while values exceeding 20 ceased to induce changes in the filtered signal. It can be observed that the experimental pressure measurements in laminar, transition and turbulent flow differ due to the different characteristics of each flow regime. Low Reynolds numbers (laminar, Re≤128) are grouped together with static fluid (Re=0), then high Reynolds numbers (turbulent, 17182≤Re≤45,955) and finally moderate Reynolds numbers (transition, 1160≤Re≤8591). It is noteworthy that comparable results were achieved for Sensors 2 and 4 (not shown here).

In the static and laminar flow regimes (Re≤128), the average pressure values exhibit higher magnitudes compared to those in the transitional and turbulent regimes. This discrepancy can be attributed to variations in experimental conditions (see Table 1): experiments conducted at Re≤128 utilized mass glucose concentrations of 20% with a fluid density of 1110.8 kg · m^−3^, while those in the transitional and turbulent regimes were performed using distilled water with a fluid density of 998.7 kg · m^−3^. Consequently, the hydrostatic pressure exerted by the liquid column at Re≤128 was greater.

In laminar regime, the interaction between the flow around the Rushton impeller and the existing flow around the baffle (if present) is minimal [29]. Therefore, it is expected that the pressure values and pressure fluctuations in this flow regime approach those found in a static fluid (Re=0), consistent with the trend observed in Figure 11.

As the Reynolds number is increased, the transition from laminar to turbulent flow occurs over a wide range of Re with significant impeller–baffle interaction. The decrease in pressure measurements in the transition with respect the turbulent flow regime can be explained by the phenomenon of pressure drop caused by turbulence generated by agitation. Turbulence increases internal friction within the fluid and causes energy loss due to flow resistance. This is similar to the phenomenon of pressure drop in a pipe due to fluid friction against the pipe wall [31]. This decrease in total pressure does not necessarily mean that the absolute pressure in the tank is decreasing, but rather that the pressure measured at the baffles or specific points in the tank where the sensors are located is lower in the transitional flow regime than in the turbulent regime.

The transitional flow regime is characterized by a mixture of laminar and turbulent flow patterns with intermittent fluctuations and eddies. The presence of instabilities in this flow regime has been reported, leading to the appearance of spurious frequencies [29] and occasional fluctuations in velocity and pressure [32]. Thus, local pressure measurements for the transition flow may be more variable than in turbulent flow. This explains why the pressure measurements in the transition and turbulent flow are different in magnitude and in the frequency of their fluctuations.

**Figure 12:**
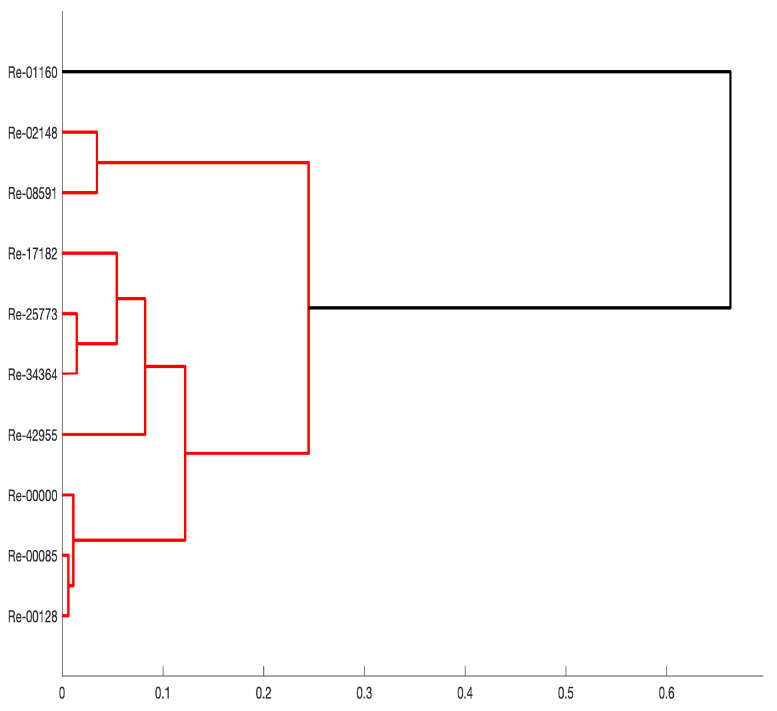
Hierarchical bottom-up clustering for sensor 1 for the eleven examined Reynolds numbers.

In order to group the values of the force sensors used (each value corresponding to a Reynolds number), a machine learning algorithm called Hierarchical Agglomerative Clustering (HAC) was applied. The principle of HAC is to group two elements at a time, i.e., starting by grouping the two closest elements. Subsequently, elements and groups, or groups and groups, are successively merged. The algorithm ends when all elements are grouped into a single cluster. The HAC analysis is shown in Figure 12. As can be seen in this figure, and in agreement with the previous discussion, there is an ordering according to the Reynolds flow regime: transition (top, Re of 1160, 2148 and 8591), static or laminar (bottom, Re of 0, 85 and 128) and turbulent (between top and bottom, Re of 17,182, 25,773, 34,364 and 42,955).

## 5. Conclusions

Pressure fluctuations within a mixing tank offer crucial insights into the prevailing flow regime. An experimental apparatus designed to study pressure fluctuations within a mixing tank is presented. The results presented in this study were generated using a Rushton turbine, categorized as a radial flow impeller. The force sensors were strategically positioned in the baffle region, tracking the flow induced by the Rushton impeller. The system has been conceived, designed and constructed in all its constituent electronic parts. In addition, software for wireless communication with a personal computer has been successfully developed.

An innovative experimental prototype, the Monitoring and Acquisition System for Pressure Fluctuations in Stirred Tanks (MASPFST), has been designed and implemented. The system facilitates the characterization of flow regimes in mechanically stirred tanks by monitoring pressure fluctuations, eliminating the necessity for real-time knowledge of viscosity and density.

The MASPFST prototype uses Force Resistive Sensors strategically placed on the baffles inside the mixing tank. These sensors collect pressure data, which are then processed and transmitted to a computer via an electronics module based on an Arduino UNO development board. This data can be visualized in real time or stored for later analysis, providing researchers with valuable information about the flow regime during the mixing process. In addition, an analysis module within the system can determine the operational flow regime.

Experimental tests conducted with our prototype demonstrated its versatility, successfully capturing real-time variations in Reynolds numbers ranging from static, laminar, transition to fully turbulent flow, i.e., 0≤Re≤ 42,955. The data obtained from our system showed different pressure values corresponding to different flow regimes.

In our literature review, no previous study has been found related to the real-time identification of flow regimes in mechanically stirred tanks using force sensors. Therefore, the MASPFST could become a valuable tool for researchers in this field. Thus, it represents a novel and practical solution for identifying flow regimes in stirred tanks, providing valuable insights for optimizing industrial mixing processes. This research contributes to the growing body of knowledge in the field of mixing operations and promises to improve process efficiency and quality in various industrial applications.

It is important to note that outcomes obtained with an axial flow impeller, such as a pitched blade or high-efficiency impeller, may significantly differ from those observed in our investigation with a radial flow impeller. Consequently, further studies are warranted to explore the ideal locations for force sensors when employing axial flow impellers.

## Figures and Tables

**Table 1:** Pinouts on the Arduino UNO board.

Signal	Terminal	Type
Voltage 5 V DC.	5 V DC.	Output
Ground	GND	—
Ground	GND	—
Voltage 9 V DC	Vin	Input
Sensor 1	A0	Input
Sensor 2	A1	Input
Sensor 3	A2	Input
Sensor 4	A3	Input
Sensor 5	A4	Input
Serial port	TX	Output
Serial port	RX	Input
Ground	GND	—

**Table 2:** Initializing parameters for the Arduino UNO board.

Parameter	Usage
int sensorPin1 = A0;	Analog Input Sensor 1
int sensorPin2 = A1;	Analog Input Sensor 2
int sensorPin3 = A2;	Analog Input Sensor 3
int sensorPin4 = A3;	Analog Input Sensor 4
int sensorPin5 = A4;	Analog Input Sensor 5
boolean KEY = false;	Flag for sending data
int mensaje = 0;	Data reading flag
void setup () {Serial.begin (9600);}	Communication at 9600 bps

**Table 3:** Configuration settings.

Parameter	Value
Port	COM-8
Baud Rate	9600
Sensor	4 sensors
Name file	RowData.xls

**Table 4:** Properties and Reynolds number covered of the fluids considered in this study at 20 °C.

Fluid	Density (kg · m^−3^)	Viscosity (Pa·s)	Reynolds Covered	Fluid Characteristics
1	1110.80	0.0282	Re≤128	Static and extended laminar flow
2	998.70	0.001	1160–42,955	Turbulent transition to fully turbulent flow

## Data Availability

The data that support the findings of this study are available from the corresponding author upon reasonable request. The data are not publicly available due to authors are concerned that they may be misinterpreted if made public without proper context or supervision.
